# Supercritical Impregnation of Ketoprofen into Polylactic Acid for Biomedical Application: Analysis and Modeling of the Release Kinetic

**DOI:** 10.3390/polym13121982

**Published:** 2021-06-17

**Authors:** Lidia Verano Naranjo, Cristina Cejudo Bastante, Lourdes Casas Cardoso, Casimiro Mantell Serrano, Enrique José Martínez de la Ossa Fernández

**Affiliations:** Chemical Engineering and Food Technology Department, Faculty of Science, Wine and Agrifood Research Institute (IVAGRO), University of Cadiz, 11519 Puerto Real, Spain; lidia.veranonaranjo@alum.uca.es (L.V.N.); lourdes.casas@uca.es (L.C.C.); casimiro.mantell@uca.es (C.M.S.); enrique.martinezdelaossa@uca.es (E.J.M.d.l.O.F.)

**Keywords:** supercritical solvent impregnation, drug release, polylactic acid, ketoprofen, swelling, drug loading, drug delivery implants

## Abstract

Ketoprofen (KET) is an anti-inflammatory drug often used in medicine due to its analgesic and antipyretic effects. If it is administered in a controlled form by means of different dosing devices, it acts throughout the patient’s recovery period improving its efficacy. This study intends to support the use of supercritical solvent impregnation (SSI) as an efficient technique to develop polylactic acid (PLA) functionalized with ketoprofen, for use as controlled drug release devices. For this purpose, firstly, the influence of different SSI variables on the desirable swelling of the polymer structure, while avoiding their foaming, were evaluated. Then, the resulting ketoprofen loading was evaluated under different pressure/temperature conditions. It was generally found that as pressure and temperature are higher, the drug impregnation loads also increase. The maximum impregnation loads (at about 9% KET/PLA) were obtained at 200 bar and 75 °C. In vitro drug release tests of the impregnated compound were also carried out, and it was found that drug release profiles were also dependent on the specific pressure and temperature conditions used for the impregnation of each polymer filament.

## 1. Introduction

The use of biocompatible polymeric devices loaded with drugs or bioactive compounds is growing, and it is seen as an excellent alternative to conventional implants or to other clinical therapies. These devices allow to locally and gradually release a particular compound of interest for an improved treatment of certain disorders. They are primarily made out of bioabsorbable polymers, which disintegrate and are absorbed after completing their function, without the need of any additional surgery.

The type of device and polymer to be used depends on many factors, primarily the type of drug, the dose required, and the duration of the treatment [1]. Both the loading of the polymer material with the pharmacoactive substance, and the subsequent release of such substance once the device has been implanted in the patient’s body, are complex processes requiring a number of correlations between the polymer, the drug, and the medium. It is, therefore, important to complete a number of research studies to gain some insight into such correlations and their effects.

Polymeric matrices are generally functionalized either by submerging them for a certain period of time into a solution that contains the drugs or active compounds to be impregnated, or, alternatively, by adding the substances of interest during the polymer synthesis process. Both of these methods present significant drawbacks, such as the use of toxic organic solvents, unwanted pharmacological reactions, photochemical and thermal degradation of the drugs and/or the polymers, low impregnation loads, or uneven distribution of the drug [2]. The use of certain novel techniques, such as supercritical solvent assisted impregnation or supercritical solvent impregnation (SSI) may represent a step forward to overcome some of those difficulties.

Carbon dioxide under supercritical conditions (scCO_2_) has the capacity to penetrate, plasticize and swell polymeric matrices [3]. Therefore, the impregnation of polymers with a particular substance through SSI consists of dissolving the active substance into scCO_2_ and then carrying it into the polymer. Later, as the system returns to regular atmospheric conditions, the solvent turns into gas and leaves the polymeric matrix, while the drug precipitates and remains trapped inside. Two of the advantages in this system are that the resulting impregnated device is solvent free, and also that the solute load is not limited to the outer surface of the polymer. Nevertheless, a number of factors have to be considered, such as the possible structural modifications that the polymer may undergo as a result of its own nature, the CO_2_ sorption and/or the SSI operating conditions. Such structural modifications may affect its porosity or cause a permanent swelling or foaming, which, in turn, would have a significant influence on the drug release kinetics.

Polyesters, and specially polylactic acid (PLA) in its poly-l-lactide (PLLA) and poly-d,l-lactide (PDLLA) forms, are the most widely studied polymers for the development of bioresorbable drug-delivering implantable devices. Some examples can be found in the literature regarding the supercritical impregnation of drugs or bioactive substances into lactic acid polymers shaped as films, thin discs [4,5,6], microparticles [7], or porous structures [8,9,10,11]. However, no references have been found to use supercritical impregnation on PLA filaments, despite some studies having been conducted on this type of impregnation into surgical suture materials [12,13,14]. Champeau and coworkers [14] studied the changes in the releasing profile as a function of the depressurization during the supercritical impregnation of ketoprofen in PLLA fibers. The present work intends to be a complement to Champeau’s study, and demonstrate that temperature and pressure conditions during the impregnation process have an influence on the subsequent releasing kinetics of the drug. It is, therefore, necessary not only to evaluate how the impregnation process variables influence the polymer drug loadings, but also how the operating conditions with scCO_2_ affect the permanent swelling of the polymer. By determining the degree of influence of these factors and by controlling them during the supercritical impregnation process, the carrying materials could be customized to meet drug loading and administration requirements.

On the other hand, ketoprofen (KET) is a non-steroidal anti-inflammatory drug that has been extensively used and administered in multiple forms. In fact, it has already been successfully CO_2_-assisted impregnated into polyvinylpirrilidene (PVP) microcarriers [15,16], or incorporated into hybrid aerogels composed of silica and gelatin [17] for oral administration; starch aerogels or polycaprolactone (PCL) scaffolds for bone regeneration [18]; biodegradable urethral stents made of alginate, gelatin, and gellan gum [19]; and sutures made out of PLA, lactic or glycolic acid copolymer (PLGA), polyethylene terephthalate (PET), or polypropylene (PP) [12,13,14].

In order to evaluate both the modification of the polymer physical characteristics and the efficacy of the ketoprofen supercritical impregnation into PLA filaments, this work considers a wide range of pressure and temperature. Pressures between 100 to 400 bar were chosen based on previous experience [20,21] and considering the operating limits of the equipment. A temperature range of 35–75 °C was selected to avoid structural damage in the polymer.

In addition, the subsequent release of the drugs, and how their different sample profiles fit with different mathematical models (zero-order, first-order, Higuchi, Korsmeyer–Peppas, Peppas–Sahlin, and novel multiphasic model based on the Korsmeyer–Peppas’) were evaluated. This should cast some light as to how the solute would be delivered into the patient’s body by the polymeric device, and how that delivery would vary according to the operating conditions applied during the impregnation process.

## 2. Materials and Methods

### 2.1. Raw Materials and Reactants

The 1.75 mm nominal diameter PLA filaments for the experiments were supplied by Mundo Reader, S.L. (Madrid, Spain). Ketoprofen at over 98% purity was provided by Sigma Aldrich (Steinheim, Germany) and carbon dioxide (99.99%) by Abelló Linde (Barcelona, Spain). All the other reactants used, i.e., NaOH, Na_2_HPO_4_, KH_2_PO_4_, NaCl, KCl, and HCl 37%, were of analytical grade and supplied by Panreac AppliChem (Darmstadt, Germany).

### 2.2. Polymer Supercritical Impregnation and Swelling Analyses

**Figure 1:**
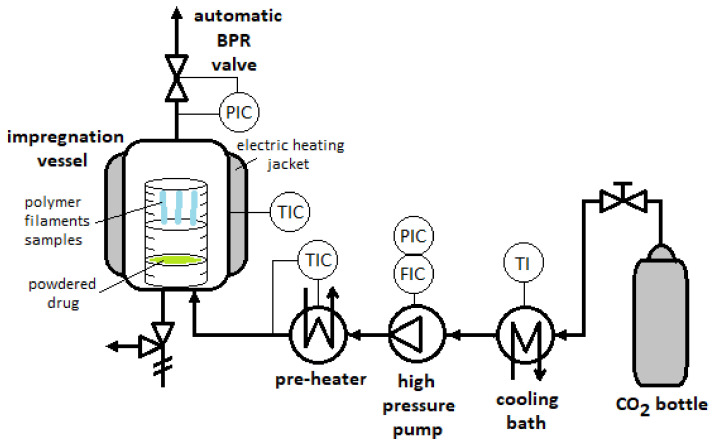
Flowchart of the supercritical solvent impregnation experiments.

The CO_2_-assisted impregnation experiments at lab scale were performaned using a high pressure SFE100 unit supplied by Thar Technologies Inc (Pittsburgh, PA, USA). A flow diagram of the experimental system is represented in Figure 1. It is mainly formed by a cooling bath, a high-pressure pump and a pre-heater for the CO_2_, a thermostatic jacketed vessel (100 mL), and a back-pressure regulator (BPR) to control the system pressure.

First of all, a series of experiments was performed in order to determine the swelling effect on the polymer by exposure to supercritical CO_2_ at different pressure and temperature conditions. A multilevel factorial design 3^2^ was followed (Table 1). For each experimental run, three pieces of PLA filament (about 3.5 cm of length and 0.1 g of weight each) were placed onto a steel support to prevent them from moving during the pressurization and depressurization steps, and that supporting device was placed inside the impregnation vessel. The vessel containing the sample was hermetically sealed, and pre-heated to the operating temperature by means of an electrical aluminum jacket. Then, the CO_2_ was pumped at 10 g/min until the desired pressure level was reached. The BPR kept the pressure constant for the preset time (30 min for the swelling analysis). After this time, the CO_2_ flow and the heating were turned off and a fast depressurization rate (100 bar/min) was applied.

The swelling effect was studied by determining the volumetric expansion of the polymer by measuring the diameter of the samples before and after the experiments (*d_o_* and *d*, respectively). Thus, volume expansion percentage could be calculated according to the following formula:

(1) $$S\;\left(\%\right)=\frac{d-d_{o}}{d_{o}}\cdot 100=\left(\;\frac{d}{d_{o}}-1\right)\cdot 100$$

Once the swelling effect on the polymers had been determined, a second set of experiments was carried out to determine the influence of the operating parameters on the supercritical impregnation of the ketoprofen into the PLA filament. The same multilevel factorial design 3^2^ (Table 1) was followed, as well as the same methodology, save a certain amount of ketoprofen which was first placed inside the impregnation vessel.

The weight of the PLA filament was approximately 0.3 g for each experiment, and the amount of ketoprofen in the vessel was calculated to be 10% over the saturation point, in order to ensure that this reactive would be present in excess throughout the process. For this estimate, the solubility values of ketoprofen in scCO_2_ at different pressure and temperature conditions were in accordance with literature references [22,23], or were also theoretically estimated following the Méndez-Santiago and Teja method [23,24]. For this set of experiments, the contact time under static conditions was 2 h in order to favor the impregnation kinetics.

The amount of impregnated drug could have been determined by gravimetry, according to the polymer weight before and after the impregnation. However, it must be noted that after the depressurization is completed, and depending on the polymer final porosity, the complete desorption of the CO_2_ may require a relatively long period (over 30 days), throughout which the weight of the whole system will slowly decrease [25]. Consequently, any method that fails to consider this fact will lead to a faulty quantification of the impregnated drug.

Therefore, for improved accuracy the amount of impregnated ketoprofen impregnated was determined by UV-Vis spectrophotometric analysis, following a similar procedure to the one proposed by Weinstein et al. [12]. Thus, different measurements were performed on the impregnated drug: (i) the first would determine the amount of ketoprofen deposited on the polymer surfaces and that would be easily released, and (ii) the second measurement would correspond to the ketoprofen that had penetrated deeper into the polymer, and which would be more gradually released into the medium. In both cases, the impregnated ketoprofen was quantified by UV-Vis spectrophotometry (Cary 60 UV-Vis, Agilent Technologies, Santa Clara, CA, USA). The measurements were carried out at 260 nm, which is the wavelength corresponding to ketoprofen highest absorbance. The concentrations of ketoprofen were determined based on the calibration curve previously calculated for a specific ketoprofen concentration range (between 1 to 15 mg/mL) in a 3 M NaOH solution.

To determine the amount of ketoprofen that had been deposited on the surface or the outer layers of the polymer, 0.1 g of the impregnated PLA filament sample was submerged into 15 mL of 3M NaOH and shaken vigorously (ca. 10 s). After that, the filament was removed, and the absorbance of the substance into the solution was measured. Subsequently, in order to quantify the ketoprofen that had been impregnated deeper into the polymer, the same filament was submerged into 25 mL of a clean NaOH solution until completely dissolved. Each test was performed twice.

The total drug loading was expressed as the mass of the impregnated ketoprofen (*m_KET_*) with respect to the mass of the PLA sample (*m_PLA_*), according to the following formula:

(2) $$Drug\;loading\;\%=\frac{m_{KET}}{m_{PLA}}\cdot 100$$

The superficial drug (*m_SUP-KET_*) was expressed as a percentage of to the total drug loading as follows:

(3) $$Superficial\;drug\;\%=\frac{m_{SUP-KET}}{m_{KET}}\cdot 100$$

### 2.3. SEM Studies

Several samples of PLA filaments, both control and impregnated ones, were observed under a Nova NanoSEM 450 microscope by FEI (Hillsboro, Oregon, USA) in order to verify the feasibility of the impregnation.

The samples had been previously coated with a thin layer of gold (15 nm) using a Cressington Sputter Coater model 208 HR from Cressington Scientific Instrument (Watford, UK) with the aim or improving their conductivity for better imaging.

### 2.4. In Vitro Release Studies

For the analysis of the ketoprofen release kinetics, a piece of impregnated PLA filament (approximately 0.05 g) was submerged into 10 mL of a phosphate buffered saline solution (PBS) according to the following composition: 1.44 g/L of Na_2_HPO_4_, 0.24 g/L of KH_2_PO_4_, 8 g/L of NaCl, 0.2 g/L of KCl, and pH adjusted to 7.4 either with NaOH or HCl. The solution containing the filament sample was kept still in a tight container at 37 °C. An aliquot of the solution was regularly taken for spectrophotometric analysis at 260 nm to determine the concentration of the released ketoprofen. The values obtained from the spectrophotometry were compared to the calibration curve previously plotted for a series of ketoprofen-PBS solutions at known concentration levels (between 1 and 16 mg/mL). Each analyzed aliquot was placed back into the same container to preserve the initial volume.

For more biologically relevant and robust data, the sink conditions had to be maintained, i.e., the release medium had to dissolve at least three times the amount of drug administered by the dosage form [26]. Hence, considering the solubility of ketoprofen in PBS at approximately 2.2 mg/mL [27], as well as the maximum amount of impregnated ketoprofen in the PLA filaments, it was confirmed that sink conditions were met.

The drug release ratio is expressed as the accumulative ketoprofen mass at a certain time *t* (*m_t_*) relative to the total mass of ketoprofen loaded (*m_KET_*) or released at infinite time (*m_∞_*):

(4) $$Drug\;released=Q_{t}=\frac{m_{t}}{m_{KET}}=\frac{m_{t}}{m_{\infty }}$$

## 3. Results

### 3.1. Supercritical Impregnation

The results obtained for permanent polymer swelling, total loading and surface loading from the supercritical impregnation of ketoprofen into PLA filaments, as well as the main experimental conclusions, are presented below.

#### 3.1.1. Swelling Effect on PLA Filaments

Under supercritical conditions, a reorganization of the polymer chains occurs as a consequence of the sorption of CO_2_. The subsequent depressurization returns CO_2_ to a gaseous state, and it escapes from the polymeric matrix while leaving behind a series of empty cavities. The increment in the free volume of the polymeric matrix may result in the permanent swelling of the polymer.

**Figure 2:**
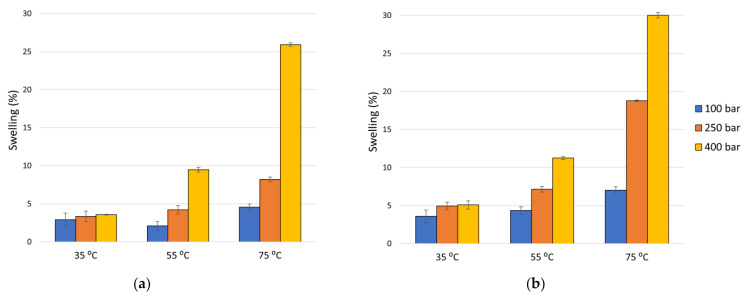
Swelling of PLA filaments, (**a**) under CO_2_ in supercritical conditions, and (**b**) under scCO_2_-assisted impregnation with ketoprofen.

The resulting permanent swelling of the PLA filaments with and without ketoprofen at different temperature and pressures conditions are shown in Figure 2.

An increase in the swelling effect was observed as the pressure was higher, being more evident at higher temperatures. Likewise, at isobaric conditions, while the temperatures increased, the swelling of the polymer was also higher. Thus, at the highest pressure and temperature conditions studied (400 bar and 75 °C) there was a large swelling and foaming effect which led to a structural deformation of the polymer that would render it impossible to be used as a biomedical implant.

**Figure 3:**
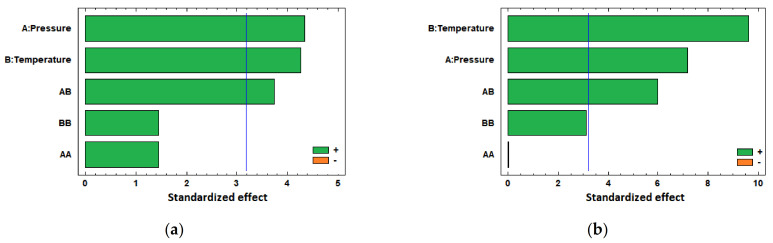
Standardized Pareto charts of the swelling of PLA filaments, (**a**) under CO_2_ supercritical conditions and (**b**) scCO_2_-assisted impregnation with ketoprofen.

These results are discussed in more detail in Section 4.1.1.; however, in general terms, it can be concluded that temperature and pressure positively significantly affect the PLA filament diameter increment when in contact with CO_2_ under supercritical conditions, regardless of the presence or absence of the drug, as can be seen in the Pareto diagrams obtained from the statistical analysis of both experimental data (Figure 3). Table 2 and Table 3 show the Analysis of Variance (ANOVA) on the experimental data of the swelling of PLA filaments under just scCO_2_, and when ketoprofen was introduced, respectively. In both cases, the *p*-values for pressure and temperature are lower than 0.05, which indicates that they are significant factors with a confidence level of 95.0%.

#### 3.1.2. Impregnation Loading of Ketoprofen into PLA Filaments

The effectiveness of the impregnation depends on the nature of the system composed by the polymer, the drug and the supercritical fluid, and the interactions between them. These interactions involve the solubility of the drug in CO_2_, the capacity of the matrix to absorb CO_2_ and swell, and the drug-polymer affinity [28]. Solubility of the drug in CO_2_, and sorption and swelling of the polymer are variable depending on the operating conditions. Thus, it is necessary to study the effect of the operating variables on the impregnation yield for each system.

**Figure 4:**
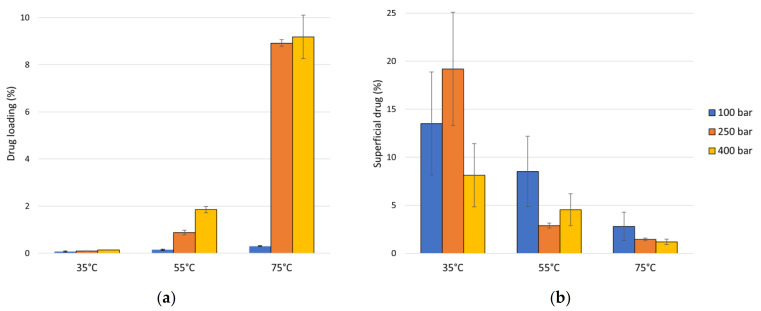
Ketoprofen impregnation loading by SSI into PLA filaments: (**a**) total drug loading: percentage of impregnated ketoprofen respect to the amount of PLA, and (**b**) percentage of superficial drug respect to the total drug loaded.

Figure 4a shows the results of ketoprofen loading into PLA filaments as a function of the operating pressure and temperature conditions. It can be seen that when both temperature and pressure are increased, the amount of impregnated drug also increases.

**Figure 5:**
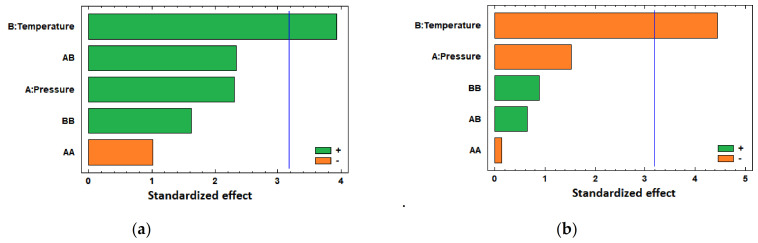
Standardized Pareto chart of the (**a**) total ketoprofen loadings in PLA filaments and (**b**) percentage of superficial ketoprofen with respect to the total amount impregnated.

The statistical analysis of these data confirms that both temperature and pressure have positive effects on the impregnated ketoprofen loadings (see Pareto chart in Figure 5a), with the effect of temperature being predominant, as can also be seen by the *p*-value lower than 0.05 for this factor in the ANOVA table (Table 4).

The maximum impregnated load is obtained at 75 °C and 250 bar with approximately 9% by mass of ketoprofen per mass of PLA. At the highest values of the conditions studied (75 °C and 400 bar) a slightly larger impregnation load was observed. However, as discussed in Section 3.1.1, the product obtained under these experimental conditions would not be considered as valid for later use due to an excessive degradation of the polymer structure.

The results from the quantification of superficial ketoprofen are shown in Figure 4b. Even if this quantification is somewhat inexact for the samples with a low level of impregnation, such as the samples impregnated at 35 °C, an assessment of the superficial impregnation of the drug on the polymer is essential to evaluate the action of the drug during the first stages of its administration, when it is expected to exhibit a higher release rate.

In general, a decrease in the amount of superficial ketoprofen is observed as either temperature or pressure are increased. The Pareto chart in Figure 5b confirms this negative effect of both variables on the superficial loading of ketoprofen, with the only relevant effect caused by temperature increments, as can also be seen by its *p*-value, lower than 0.05 (see ANOVA, Table 5).

#### 3.1.3. Optimization of the SSI of Ketoprofen into PLA Filaments

According to the statistical predictive model based on the experimental data, the maximum load and minimum swelling effect would be obtained at near 75 °C and 200 bar. Under these conditions, the experimental results would achieve a total drug loading of 8.48 ± 0.39%, with a 9.25 ± 0.38% swelling effect and 1.45 ± 0.15% of superficial drug load.

#### 3.1.4. SEM Images

**Figure 6:**
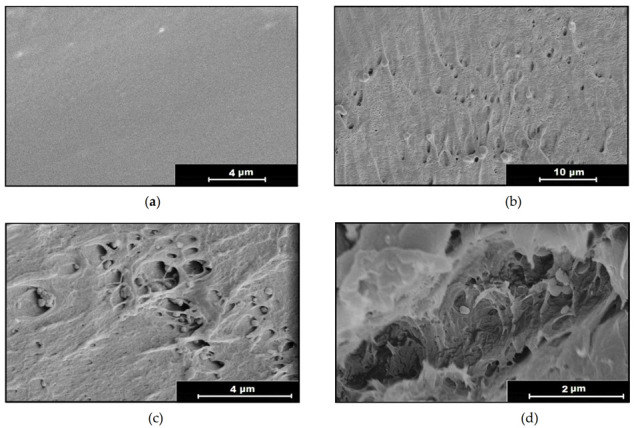
SEM images of (**a**) raw PLA filament (×1000), (**b**) PLA filament under scCO_2_ at 75 °C and 250 bar (×2400), (**c**) PLA filament impregnated with ketoprofen at 35 °C and 100 bar (×10,000), and (**d**) PLA filament impregnated with ketoprofen at 75 °C and 250 bar (×20,000). All are transversal sections of the polymer filaments.

A SEM study was performed to determine how the impregnation of ketoprofen into the PLA filaments took place. Figure 6 shows the most representative of the images, which are further commented in Section 4.1.3.

### 3.2. Drug Release Studies

**Figure 7:**
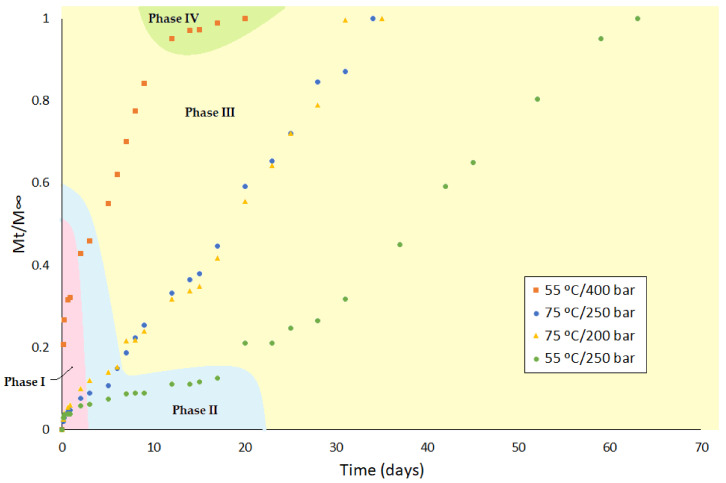
Release profiles including the different phases of the release process from the PLA filaments impregnated with ketoprofen under different conditions.

In order to determine how the different conditions during the impregnation process would affect the drug release rate, several samples that had been produced under different SSI operating conditions were selected for analysis. The samples selected had been produced at 55 °C/250 bar, 75 °C/250 bar, and 55 °C/400 bar, which were those with the higher values of drug loaded in the studied range. Additionally, the sample that had been produced under the previously established optimal conditions (75 °C/200 bar) was selected for analysis. The different release profiles obtained can be seen in Figure 7, where the different phases of the release process, which will be later on explained in Section 4.2.1, are indicated.

## 4. Discussion

### 4.1. Supercritical Impregnation Analysis

#### 4.1.1. Swelling Effect on PLA

The degree of porosity achieved by the swollen polymers, as well as the arrangement of the pores formed, are highly dependent from the operating conditions. This pore-forming effect on polymers that are intended for use in biomedicine will have different considerations depending on their target application. Thus, a high porosity is particularly interesting in the production of scaffolds, as it facilitates the compatibility with the tissues of the recipient organ. Therefore, the methods used to generate scaffolds are aimed at causing a considerable swelling of the polymer. However, in other cases, a powerful swelling effect would be considered counterproductive. For instance, when producing intraocular lenses, a high porosity would not only affect the mechanical properties of the device, but would also have other undesirable effects, such as the appearance of turbidity [29,30,31]. Likewise, in the case of vascular devices, a high degree of porosity might compromise hemocompatibility [32,33], while for controlled migration devices, high porosity would result in an undesirable early release of the drug. As the present study intends to develop a suitable method for the production of low porosity devices, a detailed analysis of this swelling effect on the polymer was crucial.

According to the results presented in Figure 2, an increase in the swelling effect was observed as the pressure was increased, at practically all of the range of temperatures tested. Other studies can be found in the literature, where the same behavior was reported for a lower temperature range. Thus, Pini et al. [34] studied the variation of the volume of powder and granular PDLLA samples in contact with CO_2_, and registered volume increments as pressure was increased (from 30 to 200 bar) while the temperature remained constant at 35 °C. Moreover, Champeau and coworkers [35] also observed an increase both in the sorption of CO_2_ and the degree of swelling of the PLLA fibers as pressure was increased up to 150 bar at 40 °C temperature.

Under isobaric conditions, an increment of the temperature would intensify the swelling effect on the polymers. However, the samples that were treated with scCO_2_ at just 100 bar while the temperature was increased from 35 until 55 °C demonstrated a lesser swelling effect. In accordance, Milovanovic et al. [5] studied the swelling pattern of PLA cylinders, which had been previously produced from melted PLA beads, under scCO_2_ at 100 bar, and they observed that temperature increments (40–75 °C) diminished the swelling effect.

When pressure and temperature conditions remain invariable, the swelling effect resulting from the action of just scCO_2_ (Figure 2a) is less noticeable than that achieved by the same process when ketoprofen is impregnated into the polymer (Figure 2b). This could be explained by the polymer’s own elasticity, which would allow it to regain its original shape to a great extent as the gaseous CO_2_ escapes from inside. However, this effect may be impeded, to some degree, by the presence of the drug. Üzer and co-workers [25] compared the swelling of polymethylmethacrylate (PMMA) rods in contact with just scCO_2_, against that resulting from the SSI of such rods with naphthalene, by measuring their longitudinal increments. They found that, for the same pressure and temperature conditions, the longitudinal increases in the samples impregnated with the chemical were greater than that of the PMMA rods in the absence of the chemical; this demonstrated that the presence of the solute positively affects the rate of CO_2_ diffusion through the interior of the polymer. Similarly, Ivanovic et al. [36] studied the production of scaffolds made of polycaprolactone (PCL) and impregnated with thymol using scCO_2_. They found that the average pore diameter in the PCL foam samples impregnated with thymol was greater than that observed in the non-impregnated PCL foam samples that had been produced under the same pressure and temperature conditions.

As temperature increases, scCO_2_ density is reduced and its diffusivity increases. On the other hand, as pressure increases, the solubility of scCO_2_ in PLA increases too, even as the diffusion coefficient recedes [37]. Both actions in turn favor the sorption of scCO_2_ into the polymer samples and the swelling effect. The absorbed carbon dioxide exerts a plasticizing effect on the polymer that is even more pronounced when the temperature rises over the polymer vitreous transition temperature, where the polymer is in an amorphous state [38]. At the same time, as the concentration of the fluid inside the polymer increases, its vitreous transition temperature, together with its melting temperature and melt viscosity, significantly decrease [39]. It should be noted at this point that the large swelling and structural deformation observed in the polymer samples that were subjected to the most extreme conditions within the studied range (400 bar and 75 °C) are a consequence of both effects: plasticization, and the resulting reduction in its melting temperature.

In those cases, when the PLA filaments were treated with just scCO_2_, it could be observed that the standardized effects exerted by pressure and temperature on the swelling are practically equivalent (Figure 3a and Table 2); however, when the drug comes into play (Figure 3b and Table 3), the effect of temperature stands out over that of pressure, as temperature has a more significant influence on the mass transfer phenomena.

#### 4.1.2. Impregnation Loading of Ketoprofen into PLA Filaments

For the same pressure value, the increase in temperature also caused an increase in the total drug loading, as can be seen in Figure 4a.

There is a crossover pressure, above which an increase in temperature produces an increment of the solubility, while below this pressure, the temperature increments negatively affect solubility. Sabegh et al. [23] studied the solubility of ketoprofen at temperatures between 35 and 65 °C and pressures between 150 and 400 bar, determining that the solubility of the drug increases with increasing temperature and pressure. They also found that, under the studied conditions, the solubility of ketoprofen remained above the crossover region. As the pressure and temperature conditions studied in the present work are above the conditions studied by Sabegh and coworkers, it can be assured that they are above the crossover region and, therefore, as the temperature increases, the solubility of ketoprofen in scCO_2_ will also increase.

On the other hand, at the pressure levels tested in this study, an increase in temperature leads to a decrease in the density of scCO_2_, which favors the diffusion of the fluid towards the inside of the polymeric matrix. Furthermore, as shown in Figure 2, an increase in temperature generally increases the swelling of the polymeric matrix, which also promotes impregnation.

For the same temperature value, as the pressure increases, the impregnated ketoprofen loading increases, as can be seen in Figure 4a. As the pressure is above the crossover point, an increase in pressure means an increase in the solubility of ketoprofen in the supercritical fluid, which favors the mass transfer. However, an increase in pressure also causes an increment in the density of the supercritical CO_2_, which could negatively affect the diffusion process of the fluid into the polymer. On the other hand, as seen in Figure 2, a higher pressure level promotes the swelling of the polymer and greater impregnation loads. By balancing these factors, a certain increase in pressure would increase the amount of drug impregnated.

In agreement with these analyses, Champeau et al. [14] also observed an upward trend in drug loading with increasing pressure (100–350 bar) and temperature (40–90 °C) for the supercritical impregnation of ketoprofen into PLLA surgical sutures.

Regarding the amount of ketoprofen on the polymer surface, according to the experimental data, it generally decreased as either temperature or pressure were increased. As previously discussed, these two variables affect the mass transfer factors, so that when both increase, supercritical impregnation deeper into the polymer is more efficient, thus decreasing the relative superficial load of the drug. The degree of porosity and the configuration of the cavities are likely the most important factors affecting the penetration of the solute into the polymeric matrix during the supercritical impregnation process.

No literature studies have been found to deal with the correlation between the superficial load of the solute and the values of the variables that operate in supercritical impregnation of similar systems. Although Weinstein et al. [12] measured the superficial impregnation of ketoprofen in PLGA sutures, in their work they only mentioned that the values obtained for the superficial impregnation of ketoprofen were below 4% of the total ketoprofen loading for all of the operating conditions studied.

#### 4.1.3. SEM Analysis

Figure 6a shows a transversal image of a PLA filament before its processing, where a relatively smooth surface with some of the characteristic imperfections of this material, as well as those caused by the cutting of the sample, can be observed. Figure 6b shows an image of the same PLA filament after being subjected to supercritical CO_2_ at 75 °C and 250 bar, where, unlike the previous image, a rough surface, with some pores or channels that have been opened by the diffusion of the CO_2_ inside the polymer and that seem to reach a size of ~1 μm, can be observed.

Figure 6c shows the PLA filament impregnated with ketoprofen at 35 °C and 100 bar, where, in spite of these being the conditions that produced the poorest impregnation results (less than 0.1% with respect to polymer mass) and almost no swelling, the impregnated drug particles are perfectly visible, as well as some similar pores to those observed in Figure 6b, which is in agreement with the swelling values that can be seen in Figure 2. The sample in Figure 6d, impregnated with ketoprofen at 75 °C and 250 bar, shows a high impregnation efficacy (around 9% with respect to polymer mass). It can be seen in this image that a high degree of porosity was achieved under these conditions, with ~5 μm diameter cavities, which is again in agreement with the values presented in Figure 2. It is worth noting that in these samples the impregnated solute particles are less visible than those impregnated into the polymer under less favorable conditions. This could be explained by the greater dispersion displayed by the ketoprofen when a larger free volume is available inside the polymer.

### 4.2. Ketoprofen Release Profiles

#### 4.2.1. Analysis of the Ketoprofen Release Kinetics

The apparently straightforward drug release process is affected by multiple and complex factors and their interactions. Chemical composition, structure of the drug and the polymer, geometry of the polymeric system, swelling capability of the polymer in contact with the release liquid, polymer degradation, solubility of the drug, diffusion capacity of both the drug and the release medium into the polymeric matrix, pH, and temperature are some such factors, among others. Nevertheless, solute diffusion and polymer degradation are the two most prominent parameters in biodegradable drug delivery systems to determine the way the drug is released [40]. In this case, the differences in the porous structures of the PLA filaments, resulting from the different temperature and pressure conditions applied for their SSI with ketoprofen, had not only affected the diffusion on the solute into the polymer, but would also have a role in the releasing of the drug into the medium.

A system whose substance release pattern is entirely governed by Fickian diffusion will present a release profile similar to the square root function, whereas when the degradation of the polymer is the driving force, the release profile will be linear in time (zero-order). However, this type of polymer rarely presents a mono-phasic release profile; rather, biphasic or tri-phasic profiles are more commonly found. The typical tri-phasic release profile would ideally have a sigmoidal graphical shape. Nevertheless, when all the phases are present, the release profile will vary according to the amount of drug released and the duration of each phase [41]. The main phases that describe a multiphasic drug release process are illustrated below.

Phase I is frequently described as a burst release as, in the first moments of contact with the release medium, there is a large amount of the mass of the drug being transferred. This is due to the diffusion of the solute molecules that are in the outermost surface of the polymeric system or in the pores directly connected to its surface. At this stage, the drug concentration gradient and the device shape appear to have a greater impact on the release rate, resulting in diffusion-governed kinetics [42,43]. Figure 7 shows that the amount of drug released during this first phase by the sample impregnated at 55 °C and 400 bar (more than 40%) is much larger than that released over the rest of the experimental time (less than 10%). As previously discussed (see Figure 4b), the amount of superficial ketoprofen in this sample is greater than in the others, which would partially explain this initial rapid drug release. On the other hand, as the increase in polymer volume is an indication of its porosity level, and considering that it is somewhat higher in the sample impregnated at 55 °C and 400 bar than in the others (see Figure 2b), and given that a higher degree of porosity would imply a greater number of pores connected to the outer surface of the device, a larger initial drug release should be expected from these samples.

Phase II is a slow-release period where, as polymer hydration occurs and degradation starts, the drug slowly diffuses through the dense matrix of the polymer and the existing pores. This phase is not always present, or it is sometimes relatively short, as it is in this case. One factor that can cause this phase to be missing is that lipophilic drug molecules can efficiently diffuse through polymeric matrices [44]. On the other hand, PLA is a polymer that starts to degrade relatively soon and, in addition, the degree of porosity and the pore configuration achieved during the SSI process may accordingly speed up the hydration process of the matrix and the onset of the degradation. As a result, as can be seen in Figure 7, there is a very short and practically negligible Phase II of the sample impregnated at 55 °C and 400 bar. This phase is somewhat longer for the samples impregnated at 75 °C and relatively longer for the sample impregnated at 55 °C and 250 bar, which would indicate a decreasing level of porosity in the same order. Similarly, it can be seen in Figure 2b that the swelling percentage of the samples also decreases in this order (approximately 11% for the sample impregnated at 55 °C and 400 bar, 9% for the samples impregnated at 75 °C and 200/250 bar, and 7% for the sample impregnated at 55 °C and 250 bar).

From a medical point of view, the duration of this Phase II should ideally be as short as possible for an efficient drug administration to produce the adequate therapeutic effect. Thus, especially in the case of antimicrobial or antiviral drugs, Phase II would be critical if drug levels are to be maintained above the minimum inhibitory concentration that is required for the control of the medical condition.

Phase III is usually a period of rapid release, sometimes referred to as the second burst. With the ongoing polymer degradation, the scission of the chains becomes the dominant driving force, and the drug release profile agrees with the degradation kinetics of the supporting material. It can be seen in Figure 7 that the graphical representation of the percentage of drug released in this phase is linear with time. A steeper slope indicates a more rapid release of the drug. Thus, a rapid release can be observed from the sample impregnated at 55 °C and 400 bar, a mild delivery rate from the samples impregnated at 75 °C, and a relatively slower release from the samples impregnated at 55 °C and 250 bar. As previously mentioned, the porosity level of the samples also decreases in the same order, so that a higher porosity results in a more rapid drug release. The level of porosity and, to a greater extent, the interconnection between pores allows for a more effective hydration of the polymer as a result of the larger contact surface area.

According to the literature, a final period of slow release rate (Phase IV) may be observed at the very end of a regular tri-phasic release profile [45,46]. In this study, this final slow Phase IV could only be noticed at the end of the release cycle of the sample impregnated at 55 °C and 400 bar, which also had the shortest Phase II.

#### 4.2.2. Monophasic Models

The empirical or semiempirical classical drug release models are intended to describe mass transfer by focusing on one or two of the dominant factors previously mentioned in the kinetic analysis. Although these models may be inadequate to describe more complex systems, or systems with multiple phases, they may still provide some interesting information on the mechanisms involved. A vast array of models that analyze the release profile of a substance previously loaded into a polymer can be found in the literature. Some of them have been analyzed and applied to this study as described below.

One of the simplest ways to determine the release kinetics of a drug into a liquid is by considering that the dissolution of the solute is a kinetic process that fits to a zero-order kinetic, in which the velocity of dissolution (*dQ/dt*) reflects the amount of drug dissolved in the medium per time unit. Considering that the dissolution rate is constant and independent from time, Equation (5) allows us to calculate the amount of drug dissolved.

(5) $$Q_{t}=Q_{o}+k_{o}t$$

where *Q_t_* is the cumulative drug released at a time *t* relative to the total mass loaded (see Equation (4)), *Q_o_* is the initial drug released (usually zero), and *k_o_* is the zero-order kinetic constant.

Polymer hydrolysis can follow different kinetic orders. Generally, the simplest one is considered to be zero order and, even if the governing mechanism is the degradation of the matrix, the dissolution of the drug will also be considered to be carried out according to the same kinetics. However, some authors suggest that PLA degradation is adjusted to a first-order kinetic [47,48]. If a first order kinetic is already associated to the dissolution of the drug, the release profile would then be given by Equation (6), where *k_1_* is the first-order kinetic constant.

(6) $$Q_{t}=Q_{o}\exp\left(k_{1}t\right)$$

On the other hand, Higuchi [49] proposed a model that is mainly based on the diffusion of the solute through the matrix into the release medium. This model is based on the following assumptions [50]: (i) The matrix contains an initial drug concentration much higher than drug solubility; (ii) the diffusion is unidirectional because the edge effects are negligible; (iii) the thickness of the drug carrying device is much larger than the size of the drug molecules; (iv) the swelling or dissolution of the matrix is negligible; (v) the diffusivity of the drug is constant; and (vi) perfect sink conditions are attained in the release environment. The model equation is:

(7) $$Q_{t}=k_{H}\;t^{0.5}$$

where *k_H_* is the model constant that includes information on diffusivity, capillary, porosity, and solubility. This model has been widely and effectively used for the description of drug administration through polymeric matrices. An example, for the same type of polymer as the one discussed in this work, is the release of ramipril encapsulated in PLA and PLGA nanospheres, which was studied by Basu and coworkers [51].

The power law or Korsmeyer–Peppas model [52], a modification of the Higuchi model, is one of the most widely used drug delivery models; its equation is as follows:

(8) $$Q_{t}=k_{KP}\;t^{n}$$

where *k_KP_* is a constant that incorporates the structural and geometric characteristics of the device and *n* is an exponent that indicates the release mechanism.

This equation is widely used to describe drug release from polymeric devices, as it allows differentiation between the two main mechanisms that govern the mass transfer. The dominant mechanism will depend not only on the particular polymer, but also on its molecular structure, the shape of the device, its interaction with the drug, and the nature of the release medium, among many other aspects. For a cylindrical geometry, when the exponent *n* in Equation (5) is lower than 0.45, the drug release is controlled by a Fick´s diffusion mechanism; for values up to 0.89 the drug release rate has a practically linear dependence with time and the mechanism that controls the drug release is the polymer degradation, corresponding to zero-order kinetics or case II transport. On the other hand, for intermediate values of the exponent *n*, an anomalous transport with overlapping of both phenomena, drug diffusion and polymer degradation, should be considered [53]. Thus, it can be found systems based on PLA that, although they fit well with Korsmeyer–Peppas equation, exhibit a Fickian type of release [54]. Other systems are rather dominated by the polymer degradation phenomena [15], while there are some with a release mechanism based on the overlapping of both effects [55].

In order to quantify the contribution from either diffusion or degradation of the polymer in anomalous transport cases, the model proposed by Peppas–Sahlin should be seriously considered [56]:

(9) $$Q_{t}=k_{d}\;t^{m}+k_{r}\;t^{2m}$$

where the first term (*k_d_ t^m^*) represents the contribution through Fickian diffusion and the second term (*k_r_ t^2m^*) incorporates the case II transport, related to the relaxation or the degradation of the polymer. When compared to the power law, the coefficient *m* coincides with the given value *n* for a strictly Fickian diffusion (*m =* 0.45).

**Figure 8:**
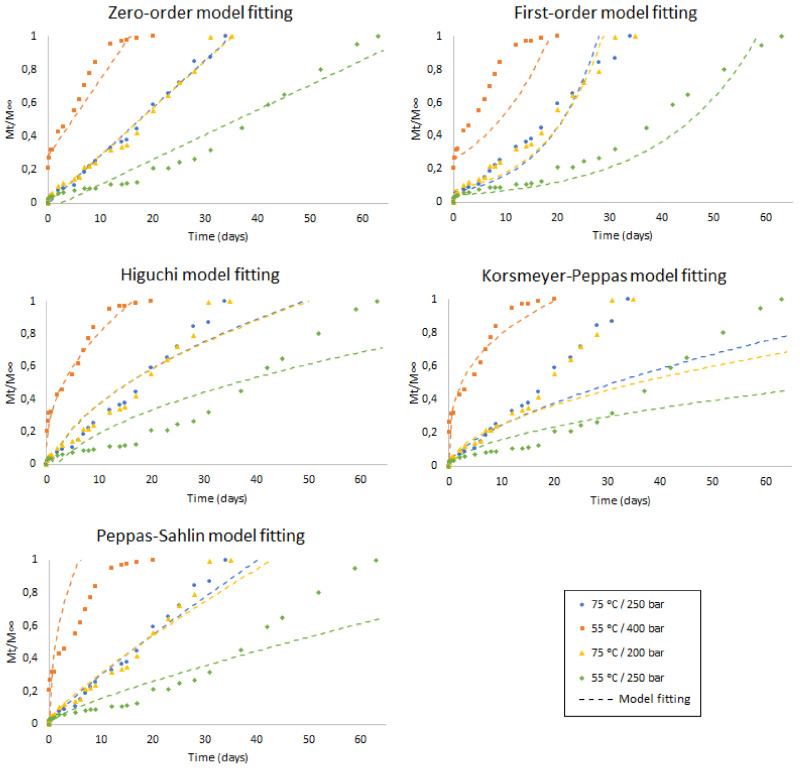
Release profiles of the different ketoprofen-impregnated PLA filament samples fit to different mathematical models.

Figure 8 shows the matching degree of the ketoprofen release experimental data with the different models analyzed under the four experimental sets of conditions considered in this study. The model parameters and R-squared values have been registered in Table 6. By observing the data obtained from each set of conditions, we can conclude that both the porosity and the swelling effect on the samples after the impregnation process play a crucial role regarding the fitting of such data with a particular kinetic model.

It can be seen from the above data that the two samples that were impregnated at 75 °C are the best fit for a zero-order release governed by polymer degradation. Thus, the higher porosity presented by the polymer after being treated with scCO_2_ under these conditions provides a degradation rate, and hence a drug dissolution rate, independent from time. The systems that display this type of release behavior are suitable for prolonged drug release as, after an initial phase, the release rate and the absorption of the drug by the host organism would be equivalent to the drug disposal rate, which in turn would result in the presence of the desired therapeutic drug levels [57]. Some authors have successfully applied this release kinetics to composite systems with a material similar to the one used in this work. For example, Abulateefeh and coworkers [58] reported a good fit to zero-order release kinetics for PLA microcapsules loaded with fluorescein sodium. Similarly, Moorkoth and coworkers [59] applied this equation to the administration of different anticancer drugs through PLLA nanoparticles.

However, the sample impregnated at 55 °C and 400 bar, which showed somewhat lower porosity values, presented initial and final diffusive phases which did not fit well with zero-order kinetics. Although their intermediate phase shows a linear release that would fit well with type of release profile, the governing mechanism of the total release is diffusion. This is evidenced by the fact that, of the four sample kinetics analyzed, this is the one that achieves the best fit with the Higuchi model, which is based on diffusion, while in the Korsmeye–Peppas model its *n* value was less than 0.45, corresponding to a Fickian diffusion release mechanism.

In contrast, the rest of the samples did not fit well with Higuchi’s model and their values for Korsmeyer–Peppas equation exponent were between 0.45 and 0.89, suggesting that the mass transfer was influenced by both diffusion of the solute and degradation of the polymer.

The sample impregnated at 55 °C and 200 bar, which showed the lowest porosity and swelling effect, did not seem to fit well to zero-order, Higuchi or Korsrmeyer–Peppas models, perhaps as its initial diffusive phase was longer, and during the polymer degradation phase the dissolution rate seemed to be more time dependent. In fact, this type of sample seems to be the best fit to a first order kinetics, although its data seem to deviate during the longer periods, where a more linear dependence with time appears. For these impregnation conditions, a first-order kinetics at the beginning of the degradation period, followed by a zero-order kinetics for the longer periods could be construed. Thus, some authors, such as Manna and coworkers [15], reported a good fit to both zero-order and first-order kinetics over the release of methotrexate from PLA- or PLGA-coated chitosan micro-implants.

Finally, it is noteworthy that none of the samples fit well with the Peppas–Sahlin model, even if similar systems, composed of PLGA copolymer nanoparticles and flurbiprofen, had been reported otherwise in the literature [60]. This lack of fit would evidence the contribution from both mechanisms to the releasing of the substance. Having said this, let us mention that the samples which had been impregnated at 400 bar and 55 °C produced a negative value of *k_r_*, which could indicate that the release of the drug was entirely Fickian. Both constants, *k_d_* and *k_r_*, obtained similar values in the rest of the experiments, which would suggest that the release was influenced by both the diffusion mechanism and the polymer degradation, perhaps consecutively and not simultaneously, as the Peppas–Sahlin model would normally indicate.

#### 4.2.3. Multiphasic Model

**Figure 9:**
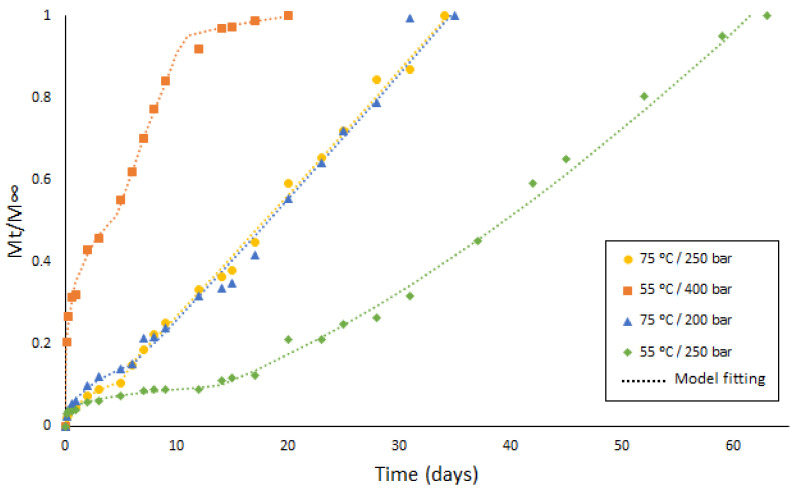
Fitting graphs of the ketoprofen-impregnated PLA filaments to the multiphasic power law model.

An experimental adjustment of the data to a multiphasic model based on the Korsmeyer–Peppas model was performed, as it would allow us to differentiate between the two main mechanisms which govern the drug release process. For this purpose, the piecewise defined function given by Equation (10) was proposed, where each function represents a consecutive interval and is defined by the power law, with the same limits for the exponent as those established by Ritger and Peppas. For its application, phases I and II were modeled with the same equation, as they represent diffusion-governed processes. Table 7 registers the adjusted parameters of this piecewise model. Figure 9 shows its fitting graphs.

(10) $$Q_{t}=\left\{\begin{array}{ccc} k_{1}\;t^{\left(n_{1}\right)} & if\;0<t\leq t_{1} & \left(phases\;I\;and\;II\right)\\ k_{2}\;t^{\left(n_{2}\right)} & if\;t_{1}<t\leq t_{2}\; & \left(phase\;III\right)\\ k_{3}\;t^{\left(n_{i}\right)} & if\;t_{2}<t\leq t_{3} & \left(phase\;IV\right) \end{array}\right.$$

It can be seen from the R-square values that the fit was quite good. The diffusion phases I and II are expressed according to an exponential release profile, where exponent *n_1_* is lower than 0.45. The kinetic constant *k_1_* depends on the amount of drug impregnated on the outermost surfaces of the polymer, i.e., the amount of drug that can easily diffuse. The kinetic constant of the samples impregnated at 400 bar is much higher than those of the other samples, and the calculated amount of superficial ketoprofen on these samples is also larger than that on the other samples (see Figure 4b). Hence, the fraction of drug released during phases I and II (Q_1_) from the sample that had been impregnated at 400 bar was greater (more than 50%) than that registered for the other three samples (about 10%). This could be so due to the larger pore size and a greater number of interconnections between them in the 400 bar samples, allowing a more rapid transfer of the drug into the medium even under a higher pressure level.

The duration of this first diffusive stage was approximately 5 days for three of the experiments and approximately 12 days for the sample impregnated at 250 bar and 55 °C. This difference could be attributed to the low degree of porosity and reduced pore size reached in the impregnation stage under mild conditions. In this case, the diffusion of the drug, even from a relatively shallow impregnation inside the filament, through a relatively denser polymer matrix, would face more difficulties.

The degradation of the polymer takes place in Phase III. The different porosity levels reached by the polymer as a result of its supercritical impregnation influenced both the pattern and the kinetics of the polymer degradation and, consequently, its release rate. The sample that had been impregnated at 400 bar and 55 °C had an exponent *n_2_* between 0.45 and 0.89, suggesting an anomalous transport case that would affect drug release, and where mass transfer occurs simultaneously by diffusion and by polymer degradation. Regarding the other three experiments, values for *n_2_* greater than 0.89 were obtained, which indicate that polymer degradation is the governing mechanism for the solute release. This exponent value, which was very close to the unit for the two samples impregnated at 75 °C, fits closely with a zero-order release kinetics. As in previous phases, the release of the drug impregnated under mild conditions was slower, which suggests that its degradation was also slower due to the polymer low porosity level.

As already discussed in Section 4.2.1, Phase IV tends to appear when Phase II is very short. It can be seen from the graph that the Phase II of the sample that had been impregnated at 400 bar is very short, or practically non-existent, and it is the only sample that displays this slow Phase IV at the end of the release period.

## 5. Conclusions

Pressure and temperature conditions for the supercritical impregnation of ketoprofen into PLA filaments determine not only the amount of drug impregnated, but also the permanent degree of swelling of the polymer after the impregnation process, an effect that is correlated with its porosity. In general, as pressure and temperature increase, both the amount of impregnated drug and the swelling of the polymer increase, with a more prominent influence from temperature on mass transfer phenomena.

In addition, the degree of porosity, as well as the arrangement of the pores, determines the drug release pattern exhibited by the impregnated filaments. Thus, by modulating the operating conditions over the supercritical impregnation, customized supporting materials could be obtained to suit the desired total dose and drug delivery profiles that would meet each particular medical treatment requirements.

## Figures and Tables

**Table 1:** Experimental design to swell and impregnate PLA with ketoprofen using scCO_2_.

	Fixed Parameters		Varying Parameters		Response
Swelling study	CO_2_ flow until target pressure Contact time Depressurization rate Amount of PLA	10 g/min 0.5 h 100 bar/min 0.3 g	Pressure Temperature	100, 250, 400 bar 35, 55, 75 °C	Swelling
SSI	CO_2_ flow until target pressure Contact time Depressurization rate Amount of PLA Amount of KET	10 g/min 2 h 100 bar/min 0.3 g Saturation	Pressure Temperature	100, 250, 400 bar 35, 55, 75 °C	Swelling Drug loading

**Table 2:** ANOVA table showing the swelling of PLA filaments under supercritical conditions.

	Df	Sum Sq	Mean Sq	F-Value	*p*-Value
A: Pressure	1	144.354	144.354	18.90	0.0225
B: Temperature	1	138.913	138.913	18.19	0.0236
AA	1	15.9989	15.9989	2.10	0.2436
AB	1	107.33	107.33	14.06	0.0331
BB	1	15.9989	15.9989	2.10	0.2436
Error	3	22.9088	7.63626	-	-
Total	8	445.503	-	-	-

R-square = 94.8578%.

**Table 3:** ANOVA table presenting the swelling of PLA filaments after the SSI with ketoprofen.

	Df	Sum Sq	Mean Sq	F-Value	*p*-Value
A: Pressure	1	165.585	165.585	51.53	0.0056
B: Temperature	1	297.229	297.229	92.50	0.0024
AA	1	0.00269	0.00269	0.00	0.9787
AB	1	115.348	115.348	35.90	0.0093
BB	1	31.0209	31.0209	9.65	0.0530
Error	3	9.63964	3.21321	-	-
Total	8	618.825	-	-	-

R-square = 98.4423%.

**Table 4:** ANOVA table including the total ketoprofen loadings in PLA filaments.

	Df	Sum Sq	Mean Sq	F-Value	*p*-Value
A: Pressure	1	18.9038	18.9038	5.36	0.1034
B: Temperature	1	54.5414	54.5414	15.48	0.0292
AA	1	3.65401	3.65401	1.04	0.3835
AB	1	19.404	19.404	5.51	0.1006
BB	1	9.31681	9.31681	2.64	0.2024
Error	3	10.5708	3.52358	-	-
Total	8	116.391	-	-	-

R-square = 90.9179%.

**Table 5:** ANOVA table presenting the percentage of superficial ketoprofen achieved through the SSI of PLA filaments.

	Df	Sum Sq	Mean Sq	F-Value	*p*-Value
A: Pressure	1	19.9838	19.9838	2.30	0.2266
B: Temperature	1	171.521	171.521	19.74	0.0212
AA	1	0.166272	0.166272	0.02	0.8987
AB	1	3.59102	3.59102	0.41	0.5660
BB	1	6.82036	6.82036	0.79	0.4409
Error	3	26.0642	8.68805	-	-
Total	8	228.147	-	-	-

R-square = 88.5757%.

**Table 6:** Model parameters and R-squared values obtained from the fitting of the ketoprofen-impregnated PLA filament samples to the different mathematical models.

		Impregnating Conditions			
		55 °C, 400 bar	75 °C, 250 bar	75 °C, 200 bar	55 °C, 250 bar
Zero-order	$R^{2}$	0.8780	0.9936	0.9824	0.9452
	$k_{O}$	0.0459	0.0287	0.0282	0.0149
First-order	$R^{2}$	0.8162	0.8484	0.8668	0.9536
	$k_{1}$	0.0735	0.1022	0.0919	0.0551
Higuchi	$R^{2}$	0.9747	0.9002	0.8781	0.7775
	$k_{H}$	0.2207	0.1640	0.1610	0.1089
Korsmeyer–Peppas	$R^{2}$	0.9625	0.9757	0.9737	0.8012
	$k_{KP}$	0.3786	0.0591	0.0737	0.0449
	n	0.3224	0.6219	0.5367	0.5571
Peppas–Sahlin	$R^{2}$	0.5533	0.8818	0.7674	0.4846
	$k_{d}$	0.4571	0.0123	0.0317	0.0158
	$k_{r}$	−0.0587	0.0337	0.0282	0.0147

**Table 7:** Model parameters and R-squared values obtained from the fitting of the ketoprofen-impregnated PLA filaments to the multiphasic power law model proposed in this work.

		Impregnating Conditions			
		55 °C, 400 bar	75 °C, 250 bar	75 °C, 200 bar	55 °C, 250 bar
Phases I & II	$R^{2}$	0.9970	0.9949	0.9933	0.9885
	$k_{i}$	0.3590	0.0526	0.0696	0.0503
	$n_{i}$	0.2348	0.4491	0.4424	0.2501
	$t_{i}\;\left(days\right)$	5	5	5	12
	$Q_{i}$	0.55	0.11	0.14	0.09
Phase III	$R^{2}$	0.9991	0.9943	0.9904	0.9938
	$k_{i}$	0.1682	0.0231	0.0214	0.0016
	$n_{i}$	0.7328	1.0659	1.0856	1.5587
	$t_{i}\;\left(days\right)$	14	34	35	63
	$Q_{i}$	0.97	1.00	1.00	1.00
Phase IV	$R^{2}$	0.9917			
	$k_{i}$	0.7807			
	$n_{i}$	0.0829	_	_	_
	$t_{i}\;\left(days\right)$	20			
	$Q_{i}$	1.00			

## Data Availability

All the data presented in this study are available in this article.

## References

[1] Davis S.S. (2000). Drug delivery systems. Interdiscip. Sci. Rev..

[2] Braga M.E.M., Pato M.T.V., Silva H.S.R.C., Ferreira E.I., Gil M.H., Duarte C.M.M., de Sousa H.C. (2008). Supercritical solvent impregnation of ophthalmic drugs on chitosan derivatives. J. Supercrit. Fluids.

[3] Kazarian S.G. (2000). Polymer Processing with Supercritical Fluids. Polym. Sci..

[4] Yu J.P., Guan Y.X., Yao S.J., Zhu Z.Q. (2011). Preparation of roxithromycin-loaded poly(l-lactic acid) films with supercritical solution impregnation. Ind. Eng. Chem. Res..

[5] Milovanovic S., Kuska R.M., Škorić-Lučić M.L., Kalagasidis-Krušić M.T., Frerich S., Žižović I.T., Ivanović J.Z. (2016). Swelling kinetics and impregnation of PLA with thymol under supercritical CO_2_ conditions. Tehnika.

[6] Coutinho I.T., Champeau M. (2020). Synergistic effects in the simultaneous supercritical CO_2_ impregnation of two compounds into poly(L-lactic acid) and polyethylene. J. Supercrit. Fluids.

[7] Zhan S., Chen C., Zhao Q., Wang W., Liu Z. (2013). Preparation of 5-Fu-loaded PLLA microparticles by supercritical fluid technology. Ind. Eng. Chem. Res..

[8] Cabezas L.I., Fernández V., Mazarro R., Gracia I., de Lucas A., Rodríguez J.F. (2012). Production of biodegradable porous scaffolds impregnated with indomethacin in supercritical CO_2_. J. Supercrit. Fluids.

[9] Cabezas L.I., Gracia I., De Lucas A., Rodríguez J.F. (2014). Novel model for the description of the controlled release of 5-fluorouracil from PLGA and PLA foamed scaffolds impregnated in supercritical CO_2_. Ind. Eng. Chem. Res..

[10] Milovanovic S., Markovic D., Markovic A., Kuska R.M., Zizovic I., Frerich S., Ivanovic J. (2019). Supercritical CO_2_-assisted production of PLA and PLGA foams for controlled thymol release. Mater. Sci. Eng. C.

[11] Milovanovic S., Markovic D., Ivanovic J. (2019). Added-value porous materials for controlled thymol release obtained by supercritical CO_2_ impregnation process. Cell Polym..

[12] Weinstein R.D., Muske K.R., Martin S.A., Schaeber D.D. (2010). Liquid and supercritical carbon dioxide-assisted implantation of ketoprofen into biodegradable sutures. Ind. Eng. Chem. Res.

[13] Champeau M., Coutinho I.T., Thomassin J.M., Tassaing T., Jérôme C. (2020). Tuning the release profile of ketoprofen from poly (L-lactic acid) suture using supercritical CO_2_ impregnation process. J. Drug Deliv. Sci. Technol..

[14] Champeau M., Thomassin J.-M., Tassaing T., Jerome C. (2015). Drug Loading of Sutures by Supercritical CO_2_ Impregnation: Effect of Polymer/Drug Interactions and Thermal Transitions. Macromol. Mater. Eng..

[15] Manna S., Donnell A.M., Kaval N., Al-Rjoub M.F., Augsburger J.J., Banerjee R.K. (2018). Improved design and characterization of PLGA/PLA-coated Chitosan based micro-implants for controlled release of hydrophilic drugs. Int. J. Pharm..

[16] Marizza P., Pontoni L., Rindzevicius T., Alopaeus J.F., Su K., Zeitler J.A., Keller S.S., Kikic I., Moneghini M., De Zordi N. (2016). Supercritical impregnation of polymer matrices spatially confined in microcontainers for oral drug delivery: Effect of temperature, pressure and time. J. Supercrit. Fluids.

[17] Veres P., López-Periago A.M., Lázár I., Saurina J., Domingo C. (2015). Hybrid aerogel preparations as drug delivery matrices for low water-solubility drugs. Int. J. Pharm..

[18] Goimil L., Braga M.E.M., Dias A.M.A., Gómez-Amoza J.L., Concheiro A., Alvarez-Lorenzo C., de Sousa H.C., García-González C.A. (2017). Supercritical processing of starch aerogels and aerogel-loaded poly(ε-caprolactone) scaffolds for sustained release of ketoprofen for bone regeneration. J. CO2 Util..

[19] Barros A.A., Oliveira C., Reis R.L., Lima E., Duarte A.R.C. (2015). Ketoprofen-eluting biodegradable ureteral stents by CO_2_ impregnation: In vitro study. Int. J. Pharm..

[20] Bastante C.C., Cardoso L.C., Serrano C.M., Martínez de La Ossa-Fernández E.J. (2017). Supercritical impregnation of food packaging films to provide antioxidant properties. J. Supercrit. Fluids.

[21] Bastante C.C., Cardoso L.C., Fernández-Ponce M.T., Serrano C.M., Martínez de la Ossa-Fernández E.J. (2018). Characterization of olive leaf extract polyphenols loaded by supercritical solvent impregnation into PET/PP food packaging films. J. Supercrit. Fluids.

[22] Gupta R.B., Shim J.-J. (2006). Solubility in Supercritical Carbon Dioxide.

[23] Sabegh M.A., Rajaei H., Esmaeilzadeh F., Lashkarbolooki M. (2012). Solubility of ketoprofen in supercritical carbon dioxide. J. Supercrit. Fluids.

[24] Méndez-Santiago J., Teja A.S. (1999). The solubility of solids in supercritical fluids. Fluid Ph. Equilibria..

[25] Üzer S., Akman U., Hortaçsu Ö. (2006). Polymer swelling and impregnation using supercritical CO_2_: A model-component study towards producing controlled-release drugs. J. Supercrit. Fluids.

[26] (2010). Recommendations on methods for dosage forms testing. European Pharmacopoeia 8.0.

[27] Mukae K., Bae Y.H., Kim S.W., Okano T. (1990). A thermo-sensitive hydrogel: Poly(ethylene oxide-dimethyl siloxane-ethylene oxide)/poly(n-isopropyl acrylamide) interpenetrating polymer networks on-off regulation of solute release from thermo-sensitive hydrogel. Polym. J..

[28] Champeau M., Thomassin J.M., Tassaing T., Jérôme C. (2015). Drug loading of polymer implants by supercritical CO2 assisted impregnation: A review. J. Control. Release.

[29] Masmoudi Y., Ben Azzouk L., Forzano O., Andre J.M., Badens E. (2011). Supercritical impregnation of intraocular lenses. J. Supercrit. Fluids.

[30] Bouledjouidja A., Masmoudi Y., Van Speybroeck M., Schueller L., Badens E. (2016). Impregnation of Fenofibrate on mesoporous silica using supercritical carbon dioxide. Int. J. Pharm..

[31] Bouledjouidja A., Masmoudi Y., Sergent M., Badens E. (2017). Effect of operational conditions on the supercritical carbon dioxide impregnation of anti-inflammatory and antibiotic drugs in rigid commercial intraocular lenses. J. Supercrit. Fluids.

[32] Zhao J., Farhatnia Y., Kalaskar D.M., Zhang Y., Bulter P.E.M., Seifalian A.M. (2015). The influence of porosity on the hemocompatibility of polyhedral oligomeric silsesquioxane poly (caprolactone-urea) urethane. Int. J. Biochem. Cell Biol..

[33] Ahmed M., Ghanbari H., Cousins B.G., Hamilton G., Seifalian A.M. (2011). Small calibre polyhedral oligomeric silsesquioxane nanocomposite cardiovascular grafts: Influence of porosity on the structure, haemocompatibility and mechanical properties. Acta Biomater..

[34] Pini R., Storti G., Mazzotti M., Tai H., Shakesheff K.M., Howdle S.M. (2008). Sorption and swelling of poly(DL-lactic acid) and poly(lactic-co-glycolic acid) in supercritical CO_2_: An experimental and modeling study. J. Polym. Sci. Part B Polym. Phys..

[35] Champeau M., Thomassin J.M., Jérôme C., Tassaing T. (2014). In situ FTIR micro-spectroscopy to investigate polymeric fibers under supercritical carbon dioxide: CO_2_ sorption and swelling measurements. J. Supercrit. Fluids.

[36] Ivanovic J., Knauer S., Fanovich A., Milovanovic S., Stamenic M., Jaeger P., Zizovica I., Eggers R. (2016). Supercritical CO_2_ sorption kinetics and thymol impregnation of PCL and PCL-HA. J. Supercrit. Fluids.

[37] Aionicesei E., Škerget M., Knez Ž. (2008). Measurement of CO_2_ solubility and diffusivity in poly(l-lactide) and poly(d,l-lactide-co-glycolide) by magnetic suspension balance. J. Supercrit. Fluids.

[38] Goodship V., Ogur E.O. (2004). Polymer Processing with Supercritical Fluids.

[39] Kuska R.M., Milovanovic S., Frerich S., Ivanovic J. (2019). Thermal analysis of polylactic acid under high CO_2_ pressure applied in supercritical impregnation and foaming process design. J. Supercrit. Fluids.

[40] Fu Y., Kao W.J. (2010). Drug release kinetics and transport mechanisms of non-degradable and degradable polymeric delivery systems. Expert Opin. Drug Deliv..

[41] Fredenberg S., Wahlgren M., Reslow M., Axelsson A. (2011). The mechanisms of drug release in poly(lactic-co-glycolic acid)-based drug delivery systems—A review. Int. J. Pharm..

[42] Wang J., Wang B.M., Schwendeman S.P. (2002). Characterization of the initial burst release of a model peptide from poly(d,l-lactide-co-glycolide) microspheres. J. Control. Release.

[43] Wang X., Venkatraman S.S., Boey F.Y.C., Loo J.S.C., Tan L.P. (2006). Controlled release of sirolimus from a multilayered PLGA stent matrix. Biomaterials.

[44] Duvvuri S., Janoria K.G., Mitra A.K. (2006). Effect of polymer blending on the release of ganciclovir from PLGA microspheres. Pharm. Res..

[45] Bae S.E., Son J.S., Park K., Han D.K. (2009). Fabrication of covered porous PLGA microspheres using hydrogen peroxide for controlled drug delivery and regenerative medicine. J. Control. Release.

[46] Han B., Gao S.-Z., Zhang X.-H., Tian H.-B., Wang H.-T., Shang Z.-H. (2010). Preparation of aclarubicin PLGA nanospheres and related in vitro/in vivo studies. J. Appl. Polym. Sci..

[47] Gorrasi G., Pantani R., Di Lorenzo M., Androsch R. (2017). Hydrolysis and Biodegradation of Poly (lactic acid). Synthesis, Structure and Properties of Poly (Lactic Acid). Advances in Polymer Science.

[48] Goto T., Kishita M., Sun Y., Sako T., Okajima I. (2020). Degradation of Polylactic Acid Using Sub-Critical Water for Compost. Polymers.

[49] Higuchi T. (1961). Rate of release of medicaments from ointment bases containing drugs in suspension. J. Pharm. Sci..

[50] Siepmann J., Siepmann F. (2008). Mathematical modeling of drug delivery. Int. J. Pharm..

[51] Basu T., Pal B., Singh S. (2018). Fabrication of core–shell PLGA/PLA–pNIPAM nanocomposites for improved entrapment and release kinetics of antihypertensive drugs. Particuology.

[52] Korsmeyer R.W., Peppas N.A. (1981). Effect of the morphology of hydrophilic polymeric matrices on the diffusion and release of water soluble drugs. J. Membr. Sci..

[53] Ritger P.L., Peppas N.A. (1987). A simple equation for description of solute release II. Fickian and anomalous release from swellable devices. J. Control. Release.

[54] Abasian P., Radmansouri M., Jouybari M.H., Ghasemi M.V., Mohammadi A., Irani M., Jazi F.S. (2019). Incorporation of magnetic NaX zeolite/DOX into the PLA/chitosan nanofibers for sustained release of doxorubicin against carcinoma cells death in vitro. Int. J. Biol. Macromol..

[55] Das D., Das R., Mandal J., Ghosh A., Pal S. (2014). Dextrin crosslinked with poly(lactic acid): A novel hydrogel for controlled drug release application. J. Appl. Polym. Sci..

[56] Peppas N.A., Sahlin J.J. (1989). A simple equation for the description of solute release. III. Coupling of diffusion and relaxation. Int. J. Pharm..

[57] Bruschi M.L. (2015). Mathematical models of drug release. Strategies to Modify the Drug Release from Pharmaceutical Systems.

[58] Abulateefeh S.R., Alkawareek M.Y., Alkilany A.M. (2019). Tunable sustained release drug delivery system based on mononuclear aqueous core-polymer shell microcapsules. Int. J. Pharm..

[59] Moorkoth D., Nampoothiri K.M., Nagarajan S., Girija A.R., Balasubramaniyan S., Kumar D.S. (2021). Star-Shaped Polylactide Dipyridamole Conjugated to 5-Fluorouracil and 4-Piperidinopiperidine Nanocarriers for Bioimaging and Dual Drug Delivery in Cancer Cells. ACS Appl. Polym. Mater..

[60] Öztürk A.A., Yenilmez E., Şenel B., Kıyan H.T., Güven U.M. (2020). Effect of different molecular weight PLGA on flurbiprofen nanoparticles: Formulation, characterization, cytotoxicity, and in vivo anti-inflammatory effect by using HET-CAM assay. Drug Dev. Ind. Pharm..

